# Supplementary material for: Off the scale: a new species of fish-scale gecko (Squamata: Gekkonidae: Geckolepis) with exceptionally large scales
Source: PeerJ. 2017 Feb 7;5:e2955. doi: 10.7717/peerj.2955 (PMC5299998; doi:10.7717/peerj.2955)
Supplement: Supplemental Information 1 — The type locality of Geckolepis maculata Peters, 1880 [file peerj-05-2955-s006.docx]

Supplementary information

**The type locality of *Geckolepis maculata* Peters, 1880**

The taxonomic progress of recent studies has been hampered by uncertainty surrounding the type locality of *Geckolepis maculata*. This species was described by W. Peters in 1880 from a single specimen sent to him at the Museum für Naturkunde in Berlin by J. M. Hildebrandt, a German explorer and biologist who worked in Madagascar from 1878 until his death there in 1885. The holotype, while still in fairly good condition, is not accompanied by any notes by Hildebrandt himself, and Peters’ description identified its locality as ‘Anfica’, in northwestern Madagascar (Peters 1880). No location in Madagascar is today known by that name, nor has any by that name ever existed as far as we have been able to deduce. Indeed, it would be grammatically incorrect in modern Malagasy, which lacks a ‘c’, and where ‘an’ is almost never followed by an ‘f’ when at the beginning of a word, but an alternative consonant, such as a ‘t’ or a ‘k’. It must therefore have been either misspelt by Hildebrandt or mistranscribed by Peters.

Attempts to reconstruct Hildebrandt’s voyages between 1879 and June 1880 (i.e. all of his travels prior to the date when he must have sent the specimens from Madagascar) in order to narrow down the potential position of this locality have been unsuccessful; while we know much of Hildebrandt’s travels over this period (see Beentje 1998 and references therein), these have proved to be so expansive that the location could be anywhere over a range of several hundred kilometres of coast between northern and western Madagascar. Some specimens from Hildebrandt mentioned by Peters (1880) come from eastern and central Madagascar, so the shipment was clearly not specific to a single locality.

Using the fuzzyg gazeteer (http://isodp.hof-university.de/fuzzyg/) to search for place names in Madagascar similar to ‘Anfica’ reveals several alternative place names, including the morphologically and phonetically similar ‘Ambika’. There are several Malagasy towns by the name of Ambika, including one just west of Mahajanga (15.90°S, 45.80°E), which would fit the description of ‘northwestern Madagascar’ given by Peters. However, assignment of the specimen to this locality on this basis would be speculative at best.

We therefore conclude that considerable further work will be necessary to establish the provenance of ZMB 9655. Attempts to sequence its degraded DNA may yield sequences long enough to give it a genetic assignment, using modern DNA extraction methods for formalin-fixed specimens and next-generation sequencing with ancient DNA protocols. However, assuming that such attempts will be unsuccessful, a larger comparative osteological dataset is necessary to establish the degree of variation within OTUs as identified by Lemme et al. (2013). This osteological dataset, which should include a wide sampling of different OTUs, and multiple specimens from each OTU, is likely to be key to identifying the genetic group to which *G. maculata* belongs. If qualitative differences cannot be found, advanced 3D morphometric methods should be applied.

References to the Supplementary Information

Beentje HJ. 1998. J. M. Hildebrandt (1847 - 1881): notes on his travels and plant collections. *Kew Bulletin* 53:835–856.

Lemme I, Erbacher M, Kaffenberger N, Vences M, and Köhler J. 2013. Molecules and morphology suggest cryptic species diversity and an overall complex taxonomy of fish scale geckos, genus *Geckolepis*. *Organisms Diversity & Evolution* 13:87–95. 10.1007/s13127-012-0098-y

Peters W. 1880. Über die von Hrn. J. M. Hildebrandt auf Nossi-Bé und Madagascar gesammelten Säugethiere und Amphibien. *Monatsberichte der Königlich Preussischen Akademie der Wissenschaften zu Berlin* 1880:508–511.
