# Supplementary material for: Off the scale: a new species of fish-scale gecko (Squamata: Gekkonidae: Geckolepis) with exceptionally large scales
Source: PeerJ. 2017 Feb 7;5:e2955. doi: 10.7717/peerj.2955 (PMC5299998; doi:10.7717/peerj.2955)
Supplement: Appendix S1 [file peerj-05-2955-s007.docx]

Appendix 1

Measurements and meristics of the specimens examined for this study

|  | *G. megalepis* | *G. megalepis* | *G. megalepis* | *G. maculata* |
| --- | --- | --- | --- | --- |
|  | ZSM 289/2004 | ZSM 2126/2007 | ZSM 232/2016 | ZMB 9655 |
| SVL (mm) | 57.8 | 68.4 | 69.5 | 58.5 |
| TL (mm) | 71.7 | 80.1 | 74.1 | N/A |
| Ax–Gr (mm) | 23.9 | 27.2 | 32.5 | 23.7 |
| ShL (mm) | 5.0 | 5.8 | 7.0 | 6.2 |
| HL (mm) | 14.9 | 15.9 | 17.1 | 15.2 |
| HW (mm) | 13.3 | 16.1 | 18.5 | 14.2 |
| HH (mm) | 7.4 | 8.9 | 10.1 | 7.6 |
| SnL (mm) | 7.0 | 7.7 | 7.9 | 7.0 |
| ED (mm) | 4.3 | 4.3 | 4.3 | 3.7 |
| IOD (mm) | 7.7 | 8.7 | 9.6 | 7.0 |
| EED (mm) | 4.9 | 5.0 | 6.3 | 4.8 |
| IOS | 10 | 10 | 9 | 9 |
| SPL | 8/7 | 7/8 | 7 | 6 |
| IFL | 4.5 | 4.5 | 4.5 | 4.1 |
| CS | 7 | 7 | 6 | 6.0 |
| MBS | 17 | 18 | 18 | 25.0 |
| VS | 27 | 29 | 31 | 32.0 |
| VHL | 6 | 7 | 8 | 9.0 |
| L1TT | 15 | 14 | 14 | 12.0 |
| L1TF | 11 | 10 | 10 | 9.0 |
| L4TT | 18 | 21 | 20 | 17.0 |
| L4TE | 12 | 12 | 12 | 11.0 |
| SAE (mm) | 0.96 | 1.12 | 1.05 | 0.72 |
| SPE (mm) | 2.30 | 2.35 | 2.31 | 1.5 |
| mid-dorsal scale length (mm) | 4.22 | 5.36 | 5.78 | 3.1 |
