# Supplementary material for: Off the scale: a new species of fish-scale gecko (Squamata: Gekkonidae: Geckolepis) with exceptionally large scales
Source: PeerJ. 2017 Feb 7;5:e2955. doi: 10.7717/peerj.2955 (PMC5299998; doi:10.7717/peerj.2955)
Supplement: Appendix S2 [file peerj-05-2955-s008.docx]

Appendix 2

Variable osteological characters among specimens of *Geckolepis* examined.

Character description:

0. Nasal: (0) unfused, (1) fused.

1. Nasal lateral side: (0) sigmoid, (1) straight.

2. Snout: (0) pointed, (1) rounded.

3. Frontal dorsal fusion: (0) absent, (1) present.

4. Fronto-nasal suture: (0) straight, (1) wedge_shaped.

5 Frontal sculpturing: (0) absent, (1) present.

6. Frontoparietal suture (Midpoint): (0) straight, (1) sigmoid.

7. Intraparietal suture: (0) straight, (1) sigmoid.

8. Premaxillary teeth loci: (0) 13 or less. (1) more than 13.

9. Maxillary teeth loci: (0) 35, (1) 36, (2) 37, (3) 38, (4) 39, (5) 40.

10. Frontal ventral fusion: (0) absent, (1) present.

11. Parabasisphenoid rostrum: (0) absent, (1) present.

12. Fenestra premaxilla-vomer: (0) rounded, (1) notch.

13. Basicranium: (0) unfused, (1) fused.

14. Stapedial foramen: (0) absent, (1) present.

15. Infraorbital fenestra: (0) narrow, (1) broad.

16. Parietal descending process: (0) Narrow, (1) Wide.

17. Parietal contacting the crista alaris: (0) absent, (1) present.

18. Scleral ossicles: (0) 13, (1) 14.

19. Prefrontal sculpturing: (0) absent, (1) present.

20. Mental foramina: (0) 5, (1) 6.

21. Dentary teeth loci: (0) 27, (1) 28, (2) 29, (3) 30, (4) 31, (5) 32, (6) 33, (7) 34, (8) 35, (9) 36, (A) 37, (B) 38 (C) 39, (D) 40.

22. Edentulous area between the coronoid and the tooth row (pathology of ZSM 1520/2008, not symmetrical): (0) absent, (1) present.

23. Interdental space: (0) minimal, (1) well spaced.

Character scores:

*Geckolepis megalepis* ZSM 2126/2007 (FGZC 1144) 010000010101101001000A00

*Geckolepis megalepis* ZSM 289/2004 (FGZC 554) ??00???1?101??1001??1???

Note: this specimen was scanned at lower resolution than ZSM 2126/2007, and several characters could not be accurately assessed.

*Geckolepis* clade AB ZSM 1520/2008 (FGZC 1697) 110100110110011010000011

*Geckolepis maculata* ZMB 9655 011000000001101101100700

*Geckolepis humbloti* ZSM 80/2010 (FGZC 4029) [0 1]01[0 1][0 1]1010501011101?11D00

*Geckolepis humbloti* ZSM 81/2006 (FGZC 836) 0000[0 1]1010301001101010A00
