# Supplementary figures and images for: Off the scale: a new species of fish-scale gecko (Squamata: Gekkonidae: Geckolepis) with exceptionally large scales
Source: PeerJ. 2017 Feb 7;5:e2955. doi: 10.7717/peerj.2955 (PMC5299998; doi:10.7717/peerj.2955)

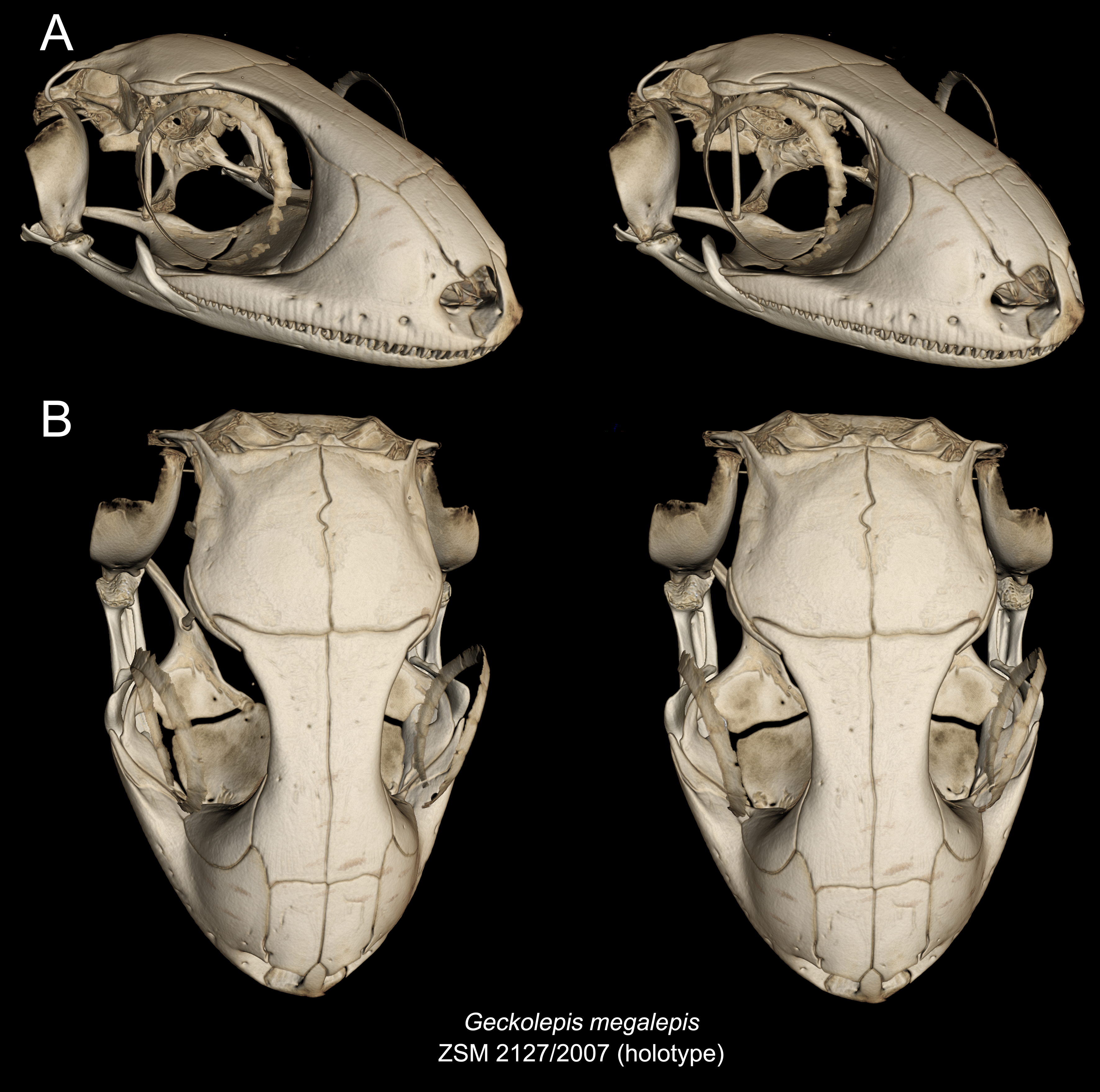

Supplement: Figure S1 [file peerj-05-2955-s009.png]
